# The Optimal Mutagen Dosage to Induce Point-Mutations in *Synechocystis* sp. PCC6803 and Its Application to Promote Temperature Tolerance

**DOI:** 10.1371/journal.pone.0049467

**Published:** 2012-11-21

**Authors:** Ulrich M. Tillich, Sandra Lehmann, Katja Schulze, Ulf Dühring, Marcus Frohme

**Affiliations:** 1 Molecular Biotechnology and Functional Genomics, Technical University of Applied Sciences Wildau, Wildau, Germany; 2 Institute of Biology, Humboldt-University Berlin, Berlin, Germany; 3 Cyano Biofuels GmbH, Berlin, Germany; Louisiana State University and A & M College, United States of America

## Abstract

Random mutagenesis is a useful tool to genetically modify organisms for various purposes, such as adaptation to cultivation conditions, the induction of tolerances, or increased yield of valuable substances. This is especially attractive for systems where it is not obvious which genes require modifications. Random mutagenesis has been extensively used to modify crop plants, but even with the renewed interest in microalgae and cyanobacteria for biofuel applications, there is relatively limited current research available on the application of random mutagenesis for these organisms, especially for cyanobacteria. In the presented work we characterized the lethality and rate of non-lethal point mutations for ultraviolet radiation and methyl methanesulphonate on the model cyanobacteria *Synechocystis* sp. PCC6803. Based on these results an optimal dosage of 10–50 J/m^2^ for UV and either 0.1 or 1 v% for MMS was determined. A *Synechocystis* wildtype culture was then mutagenized and selected for increased temperature tolerance *in vivo*. During the second round of mutagenesis the viability of the culture was monitored on a cell by cell level from the treatment of the cells up to the growth at an increased temperature. After four distinct rounds of treatment (two with each mutagen) the temperature tolerance of the strain was effectively raised by about 2°C. Coupled with an appropriate *in vivo* screening, the described methods should be applicable to induce a variety of desirable characteristics in various strains. Coupling random mutagenesis with high-throughput screening methods would additionally allow to select for important characteristics for biofuel production, which do not yield a higher fitness and can not be selected for *in vivo*, such as fatty acid concentration. In a combined approach with full genome sequencing random mutagenesis could be used to determine suitable target-genes for more focused methods.

## Introduction

There has been a revamped interest in algae and cyanobacteria biotechnology in recent years, mostly due to the possible applications for biofuel production [1], [2], [3].

As experience has shown, it is very unlikely that any form of large scale cultivation will be performed using wildtype strains. Most currently cultivated plants or animals have been extensively modified to be more suitable to application either through breeding, random mutagenesis (green revolution), and/or targeted approaches (modern GM crops).

Random mutagenesis is a useful tool to adapt stains to cultivation conditions such as the induction of tolerances. A key advantage is that only little knowledge about the underlying molecular biology is required to promote significant alterations in the phenotype [4]. It is also possible to achieve effects which require simultaneous modifications of many seemingly unrelated genes, such as those required for increased tolerance to ethanol [4]. Furthermore, when choosing the proper agent a broad spectrum of mutations can be achieved.

With the advent of next generation sequencing techniques it has become economically feasible to use random mutagenesis strategies first and then re-sequence various generated mutants. Genes of interest identified this way, could then be modified by more targeted methods.

Random mutagenesis also has a strong, but doubtful, advantage of not being classified as a method producing genetically modified organisms. In many places such as the EU this imposes fewer regulatory hurdles.

In general, mutations are classified by the type of mutation taking place and/or by the method of action by which the mutation occurs. Types of mutations are insertions, deletions and substitutions. The latter is further divided into transitions (exchange of purine to another purine or a pyrimidine to another pyrimidine), and tranversions (purine for a pyrimidine or vice versa). The mode of action is divided into two main categories: Direct mutations and indirect mutations. Direct mutations occur as a direct consequence of the mutagen, such as a miss-pairing of O^6^-Methylguanine with Thymine after treatment with Methyl methanesulphonate (or a similar alkylating agent). Indirect mutations in contrast occur as a consequence of error-prone cellular repair mechanisms such as the SOS response. Single stranded DNA which is present after mutagenesis, activates the recA protein, which in turn induces the expression of other SOS-genes. The SOS response quickly repairs DNA, but some of its repair mechanisms have a significant error rate, thereby introducing mutations into the DNA [5].

Generally, random mutagenesis is achieved by high energetic irradiation or mutagenizing chemicals. For most studies UV light or alkylation agents are the mutagens of choice. Different mutagens however have biases for the induction of certain mutations (see below). By alternating between different mutagens, the effects of these inherent biases should be reduced.

UV light induces the formation of pyrimidine dimers (mainly thymin dimers) in the DNA. These are able to cause all kinds of mutations (transitions, transversions, frameshifts, and deletions), though mainly transitions from Guanine/Cytosine to Adenosine/Thymine are observed. Deletions mainly occur in A/T rich regions [6]. Errors occurring during the nucleotide excision repair pathway (which is part of the error-prone SOS response) allow for all other mutations [6], [7], [8]. Pyrimidine dimers also lead to the formation of long single strand DNA, which activates RecA mediated homologous recombination [9], [10], [11].

Methyl methanesulphonate (MMS) is frequently used as a mutagenizing agent, since it induces all kinds of base substitutions (transitions and transversions) [12], [8]. MMS alkylates DNA, by transferring its methyl-group onto O^6^-Guanine, O^4^-Thymine N^7^-Guanine, N^3^-Adenine, N^1^-Adenine, N^7^-Adenine or N^3^-Guanine. The former two form O^6^-Methylguanine or O^4^-Methylthymine respectively, both of which no longer bind to their usual complementary base and therefore cause either a GC to TA or a TA to GC transversion. These base substitutions are direct mutation and therefore SOS independent [13], [12], [14]. However, these alkylations, as well as those on nitrogen, also cause indirect mutations, by inducing the cellular SOS response [8], [14] **.**

To increase the effectiveness of both mutagens it is important to minimize or disable the specific cellular repair mechanisms for the induced DNA lesions.

For UV light these lesions are removed mainly by the very efficient process of photoreactivation which can be easily disabled by avoiding exposure of the cells to violet/blue light (wavelengths smaller than 500 nm), required by photolyases for activation. Otherwise UV radiation has a very limited mutagenic effect [15], [7], [16], [17].

The alkylations of O^6^ or O^4^ caused by MMS are specifically repaired by O^6^-Methylguanine-DNA-Methyltransferase (MGMT). MGMT takes up the unwanted methyl-groups, thereby restoring the DNA to its usual form [18], [19], [14]. Due to the enzymatic action of the MGMT the molecule becomes irreversibly inactivated and then acts as a transcription factor for itself, inducing its own synthesis [19], [14]. It has been shown that organisms exposed to low levels of alkylating agents are more resistant to future exposures, due to an increased level of MGMT [14]. Therefore one way to minimize its effects on mutagenesis is to use short exposure times and to avoid multiple exposures within a short time frame.

In most published studies involving mutagenesis on cyanobacteria, the goal has been to create strongly aberrant mutants for physiological investigations using very harsh conditions [20], [21], [22]. Meanwhile when trying to adapt strains to cultivation conditions the goal should be to induce a high rate of point mutations, without creating strongly aberrant phenotypes and destroying whole cellular pathways.

Meireles *et al* [23] used UV mutagenesis in order to increase the yield of metabolites of interest in the microalgae *Pavlova lutheri*, however the optimal dosage was not empirically determined and the described conditions are difficult to reproduce since the UV exposure was measured in time. Ong *et al* [24] used EMS (an alkalizing mutagen) to create microalgae mutants with greatly improved growth rates at increased temperature. Chaturvedi *et al* [25] also used EMS to generate mutant microalgae. While their experiments and screening were designed to increase the content of eicosapentaenoic acid within the algae, they noted that the generated mutants demonstrated better thermotolerance compared to the WT strain. Both of these studies demonstrate the viability of using random mutagenesis to increase thermotolerance of phototrophic organisms.

For cyanobacteria the published literature on mutagenesis is far more limited. Lambert et al. [16] however did a comprehensive study comparing the effect of various mutagens on the unicellular cyanobacterium *Gloeocapsa alpicola*. They characterized each mutagen by plating cultures exposed to different dosages of mutagen on agar plates to determine the lethality and on streptomycin selection plates to determine the mutation rate.

Streptomycin inhibits cellular replication by interfering with protein biosynthesis and has been shown to directly interact with the small ribosomal subunit [26]. Resistance to Streptomycin can be conferred through various mutations in the small or the large subunit [26], [27], [28]. Within the scope of this work Streptomycin resistance is used as a marker to measure the relative rate of point mutations without discriminating the exact mutation conferring the resistance.

For this study we used *Synechocystis* sp. PCC6803 as the target organism, as it is a widely used model organism for phototrophic organisms and cyanobacteria in particular. Being from the order *Chroococcales* it is unicellular and officially classified as a fresh water strain, though it is highly tolerant to salt and marine media [29]. The Synechocystis genome was sequenced in 1996 [30], as the first genome from a photosynthetic organism.

Ultraviolet light (UV) and MMS were chosen for initial characterization with *Synechocystis* as they were determined as the best mutagens by Lambert *et al*. [16].

First the lethality and mutation induction of both mutagens on *Synechocystis* were characterized. The thus determined optimal dosages were then used for applied mutagenesis coupled with *in vivo* selection.

One big issue for the optimization of culture conditions is the temperature range, in particular when considering desert areas for outdoor cultivation. Thus we chose tolerance to high temperature as an initial target for our optimization strategy – also since it allows a relatively simple experimental setup for *in vivo* selection. Methods discussed in this paper should be easily applicable to the optimization of other characteristics however.

There have been previous studies which resulted in increased thermal tolerance of Synechocystis. This was mainly achieved by increasing the expression of heat shock proteins. Nakamoto et al. [31] demonstrated that the inactivation of the *hrcA* repressor in Synechocystis led to an increased ex of heat shock proteins and slight increase of maximal cell density when culturing at 42°C. However the ΔhrcA mutant did not have a fully induced expression of these proteins, which they speculated was due the presence of a further regulation mechanism. Suzuki et al. [32] later identified hik34 as an additional regulator for heat shock proteins. Synechocystis Δhik34 mutants, exhibited a strongly increased expression of these proteins, thus resulting in an increased thermal tolerance (surviving 3 h at 47°C whereas the wt only survived 2 h).

The random mutagenesis approach used in this paper has the advantage of not being constrained to the limited improvement of thermal tolerance achievable through increased heat shock protein expression. Thus opening the possibility to increase the thermal tolerance further than has been previously demonstrated.

## Materials and Methods

### Organism

*Synechocystis* sp. PCC 6803 salt adapted wild type, obtained from Cyano Biofuels GmbH.

### Media

All strains were cultivated in sterile mBG11 Media (30 g/l Instant Ocean®, 17,65 mM NaNO_3_, 0,18 mM K_2_HPO_4_, 0,03 mM Citric acid, 0,003 mM EDTA (disodium magnesium), 0,19 mM Na_2_CO_3_, 0,03 mM Ferric ammonium citrate and trace metals).

### UV Mutagenesis

*Synechocystis* culture (in the logarithmic growth phase) with 5*10^7^ cells/ml was placed on a petri dish (either 1 ml in a 40 mm dish, or 35 ml in a 230 mm×230 mm dish), homogeneously spread, and irradiated with up to 300 J/m^2^ UV light. Irradiation was performed in a UVC 500 crosslinker (Hoefer, San Fransisco) with only one lamp (Sankyo Denki G8T5; 8W) for a more precise dosing at a wavelength of ∼254 nm. Irradiation time was automatically adapted by the devices integrated sensor. Cells were then either used for lethality and mutation rate determination or cultivated under temperature tolerance selective conditions. Great care was given to not expose cells to any light with wavelengths <520 nm after mutagenesis, by covering cultures with a Asmetec SFG10 filter, to prevent photolyase reactivation.

### MMS Mutagenesis

*Synechocystis* culture (in the logarithmic growth phase) with 5*10^7^ cells/ml was incubated with up to 5 v% MMS (99% purity, Sigma) for 1 minute (in either a 1.7 ml reaction tube or a 500 ml centrifugation vessel). Cells were then centrifuged at 3.500 g for 1 min, the supernatant removed, and resuspended in mBG11. After a second washing step, cells were diluted and either used for lethality and mutation rate determination or cultivated under temperature tolerance selective conditions.

### Cultivation Conditions

For the mutagen characterization experiments all cultures were kept in cell culture flasks (75 cm^2^). Cultures were exposed to 4.5 µE*m^−2^*s^−1^ of light with wavelengths of >520 nm (filtered through Asmetec SFG10 filter).

For the temperature adaptation cells were cultured in a 1l bioreactor with pH-controlled 10% CO2/air addition. During a 12 h daylight phase, pH was kept at 7.3+/−0.05, light at about 125 µE/m^2^*s (170 from one side and 80 µE/m2*s from the other) and the culture was actively temperated. During the night phase cultures were bubbled with 10 ml/min air, and (passively) cooled down to 23–26°C. Cultures were diluted with fresh media so they stayed within an OD_750_ between 1 and 2 (measured with a Shimadzu UV-1800 photometer), thereby keeping them in the logarithmic growth phase.

### Determination of Lethality

The amount of viable cells after treatment was determined by plating 5*10^4^ cells on mBG11 agar immediately after the mutagenic treatment. Cells were grown with 2.2****µE*m^−2^*s^−1^ under a SFG-10 filter (to inactivate photo-reactivation) for about 2 weeks before being counted. For MMS dosages 1% and up 5*10^5^ cells were additionally plated and grown under the same conditions to increase the sensitivity of the lethality determination.

### Mutation Rate Determination

The rate of non-lethal mutations was determined by diluting the treated cells 1∶100 and cultivating them as previously described for 3 weeks, and then plating 5*10^5^ cells on selection plates (mBG11, 2 µg/ml Streptomycin). After growth on the plates for about 2 weeks with 5 µE*m^−2^*s^−1^ (without SFG10 filter), colonies were counted to determine the amount of viable cells with the ribosomal point mutation allowing for streptomycin resistance.

### Cell Counting and Viability Determination

Cells were counted either manually under a stereo microscope (SteREO Discovery V12; Zeiss, Hamburg), or using an automated procedure using the autofluorescence of phycocyanin. Fluorescence pictures of the plates where taken with a Keyence BZ9000 microscope and a phycocyanin fluorescence filter set (excitation: 600/37 nm; beam splitter: 625 nm; emission: 655/40 nm). Using the automated scanning module of the Keyence Software, pictures of an area of 25 cm^2^ where taken. Automated counting was done by an ImageJ Plugin which uses histogram thresholding for segmentation and the integrated Analyze Particles function for the counting of the colonies. The Plugin is available as Additional File S1.

To determine the vitally of the cultures after mutagenesis, cells were analyzed using a Chlorophyll viability analysis as described by Schulze et al. [33].

### Temperature Tolerance Selection

Mutagenesis and cultivation were performed as described above. For UV and MMS mutagenesis the dosages 50 J/m^2^ and 0,1 v% and 1 v% (half the culture volume per dosage) were used respectively, as they were determined to be optimal.

Mutagenesis was performed in discrete “rounds” of treatment, each consisting of three phases: exposure/selection, recovery, and determination of maximum temperature tolerance. After each round of mutagenesis three cultures were cultivated in parallel: **Backup** (previous best culture under conditions which allows good growth); **Mutant** (Mutagenized previous best culture cultivated under selective conditions) and **Control** (previous best culture, cultivated under the same selective conditions as Mutant culture). For UV mutagenesis cultures were covered with SFG10 filters for a week following the initial irradiation.

The Mutant and Control cultures were kept under highly selective conditions until the Mutant culture showed a sharp decrease in cell density (OD), spectra peaks and cell viability. Both cultures were then kept at non-selective conditions to allow surviving cells to recover. Temperature was then slowly ramped up to the new tolerated maximum-temperature. Typically the control culture did not recover and its reactor was used to test the mutagenized culture at two temperatures in parallel. A culture was considered to be growing stably at a given temperature, if it grew consistently at that temperature for at least two weeks.

## Results

### Mutagen Characterization

**Figure 1:**
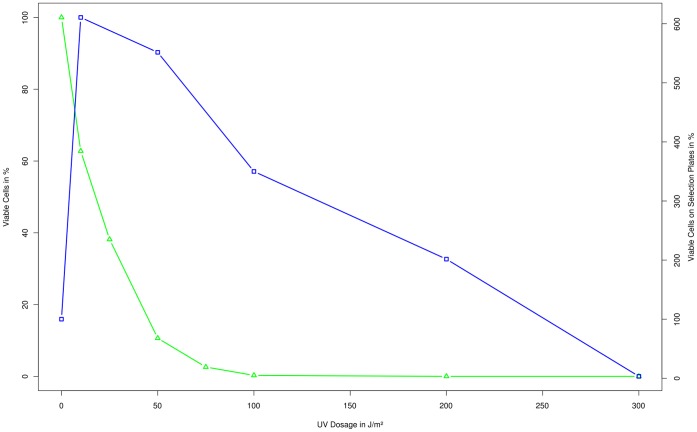
Characterization of UV. Survival rate (green; triangle) and point mutation rate (blue; square) plotted over the administered dosage of UV irradiation. The counted colonies for the survival rate and the point mutation rate were normalized by setting the control to 100%.

Characterization of UV as a mutagen for *Synechocystis* PCC6803 clearly shows an exponential decrease in the survival rate from 100% viable cells at 0 J/m^2^, to about 10% at 50 J/m^2^, and almost 0% at 100 J/m^2^ (fig. 1). The amount of viable cells on selection plates, which is equivalent to the rate of non-lethal point mutations, peaks early at 10–50 J/m^2^, indicating a high mutation rate, and then steadily drops with increased dosage.

**Figure 2:**
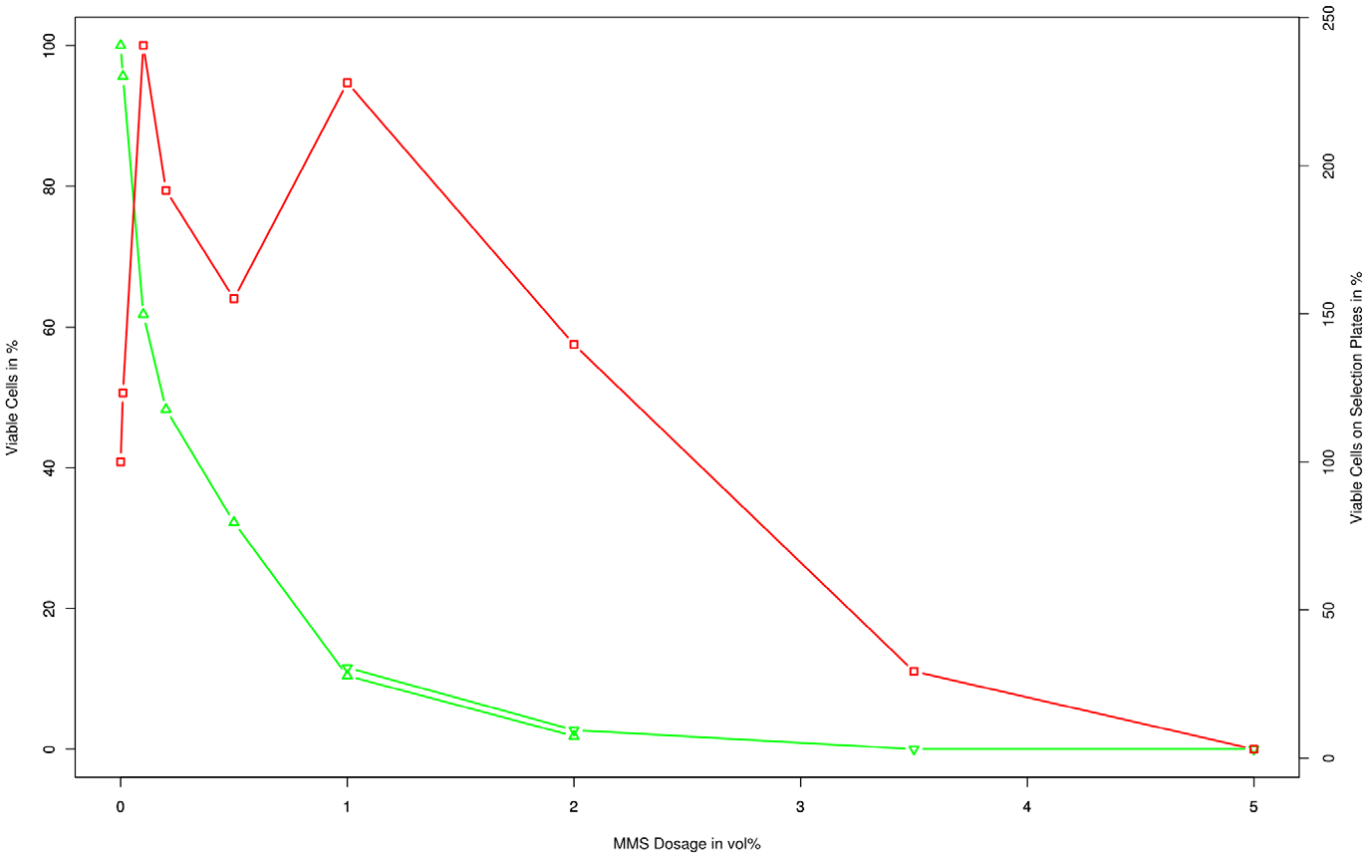
Characterization of MMS. Survival rate (green; upward triangle: 5*10∧4 cells plated | downward triangle 5*10∧5 cells plated, counted colonies divided by 10) and point mutation rate (red; square) plotted over the administered dosage of MMS exposure. The counted colonies for the survival rate and the point mutation rate were normalized by setting the control to 100%.

MMS as a mutagen for *Synechocystis* PCC6803 shows a similar exponential drop of the survival rate with increasing dose. 10% survival under our experimental condition was observed at 1 v%. MMS (fig. 2).

Surprisingly, the amount of viable cells on selection plates exhibits two distinct peaks, one at 0.1 v% and a second one at 1 v% with a significant drop in between. Beyond 1 v% the amount of colonies also steadily drops. A repetition of this experiment (data not shown) confirmed this unexpected shape of the curve.

### Applied Mutagenesis

Mutagenesis was performed in discrete “rounds” of treatment, each consisting of three phases. Within the scope of this work a round of mutagenesis is defined as the period starting with mutagen exposure of the cells and ending with the determination of the new maximum-temperature tolerated by the mutagenized culture.

Each round of mutagenesis followed the same procedure. After the mutagenesis, there was a selection phase at high temperature, where most cells were killed off. This was followed by a recovery phase at lower temperature. Once the mutagenized culture began to recover the temperature was gradually ramped up and the new tolerated maximum-temperature was determined.

In total, four rounds of mutagenesis were performed, first two rounds of UV (50 J/m^2^) and then two of MMS (0,1 v% and 1 v%). After each round of mutagenesis the temperature tolerance of the mutagenized culture was effectively increased, while the control-culture was not able to recover from the selective conditions. A summary of the applied conditions and obtained temperature tolerances can be found in table 1. In total an improvement in temperature tolerance of about 2°C could be achieved.

**Table 1:** Summary of the conditions and results of each round of mutagenesis.

Mutagenesis Round	Mutagen used	Selection temperature	Recovery temperature	Maximum tolerated temperature (day)
0 (wt)	none			<43
1	UV	44	41	43.3
2	UV	44.5	42	43.8
3*	MMS	45 (24 hours)	40	44
4	MMS	45	43	45

* Note that in the 3rd round of mutagenesis there was a technical problem with the temperation, and cultures were also temperated during the night cycle, leading to many more cells dying than usual. A recovery at lower temperature was used to rescue surviving cells.

The final culture is now able to stably grow at 45°C and 23–26°C in a 12 h day/night cycle, whereas the starting wildtype was not able to tolerate 43°C day temperature under the given conditions. The improved culture does not show an aberrant phenotype in any other aspect and most cells are still able to grow on agar plates.

At the second round of mutagenesis (UV), the viability of all three cultures (Backup, Mutant & Control) was monitored on a cell by cell basis from initial exposure until the end of the recovery phase.

**Figure 3:**
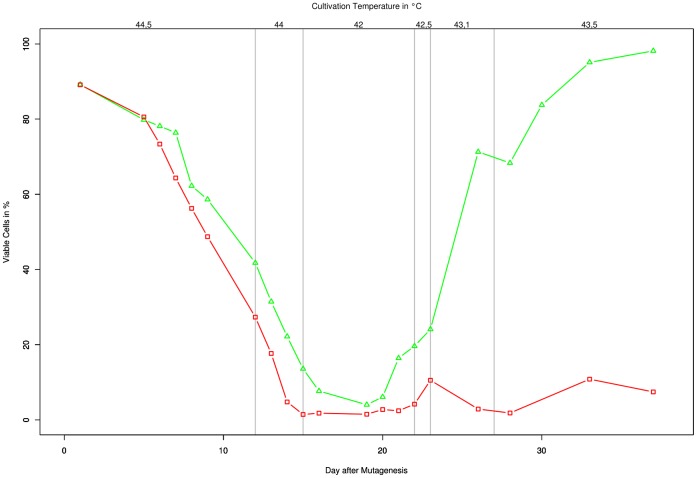
Culture monitoring after mutagenesis. The percentage of viable cells over temperature and time is shown for the mutagenized culture (green; triangle), and the control culture (red; square). The mutagenized culture was diluted to stay between OD = 1 and 2, the first dilution was necessary on day 23.

Cell viability was monitored with a novel method developed by Schulze et al. [33] which uses the Chlorophyll autofluorescence and a green fluorescence to microscopically determine the status of each cell individually. As with each round of mutagenesis, all three cultures were inoculated from the same starting culture and began with about 90% viable cells. Under the selective conditions the percentage quickly dropped off for both cultures. However during the recovery-phase at reduced temperature (42°C) the mutagenized culture was able to recover in about 5 days, whereas the amount of viable cells in the control culture stayed near 0%. As the percentage of viable cells continued to increase in the mutagenized culture the cultivation temperature was also increased in various steps (fig. 3). After growing stably at 43.5°C for 10 days the temperature was continued to be incrementally increased until the new maximum-temperature tolerance of 43.8°C was determined and the next round of mutagenesis could be performed (data not shown). The backup culture, meanwhile was constantly kept at 43.1°C (well tolerated temperature after first round of mutagenenis) and the percentage of viable cells as determined by the chlorophyll fluorescence was always between 80 and 100% (data not shown).

The course of the culture viability and temperature progression shown is representative for all rounds of mutagenesis (except the 3^rd^).

## Discussion

To characterize the mutagens MMS and UV, cells treated with varying dosages were each plated on regular agar plates to determine the survival rate and on selection plates to determine the mutation rate.

The observed exponential drop off in the survival rate for both mutagens was in line with our expectations. For both mutagens the cytotoxic effects due to DNA lesions or other chemical reactions increase in proportion with the exposure of the cells, explaining the increased lethality.

As expected, the observed rate of non-lethal point mutations induced by both mutagens was considerably increased compared to the control for both mutagens at most tested dosages. With increasing dosage however the rate of non-lethal point mutations dropped, at high dosages even below the non-mutagenized control. This can be easily explained: During cultivation in liquid media, there already was a selection for cells able to reproduce in liquid environment. Plating on selection plates additionally screens for cells still able to grow on solid media, which additionally also have a ribosomal mutation inducing a streptomycin resistance. The probability of carrying such a ribosomal mutation is increased in proportion with the applied dosage. However with increasing dosages this effect is gradually canceled out by other accumulated disadvantageous mutations which might be lethal on agar cultivation.

For UV mutagenesis the increased point mutation rate reaches its optimum for inducing non-lethal mutations between 10–50 J/m^2^. After this point the number of colonies starts dropping again, as the increased number of mutation leads to some cells unable to grow on plates at all. The rate of non-lethal point mutations for mutagenized cells however, does not drop off below the non-mutagenized control until a dosage between 200–300 J/m^2^ is reached, at which point the general survival rate was already well below 1% (fig. 1).

For MMS the number of colonies on the selection plates showed two peaks: one at 0.1 v% and one at 1 v% (fig. 2). The shape of the curve may indicate two superimposed processes. Dosages up to 0.1 v% are probably not high enough to trigger a strong response of the cellular repair mechanisms (based on MGMT), especially considering the short exposure time. As the expression of MGMT remains low, the induced DNA lesions remain unrepaired, allowing effective mutagenesis to take place. For dosages above 0.2 v%, MGMT appears to have a significant effect, lowering the overall mutation rate. At 1 v% a second local maximum is reached in spite of MGMT activity; the increased amount of alkylations appears to surpass MGMTs ability to repair them. After 1 v% the number of colonies again steadily drops off due to mutations which inhibit growth on plates, and falls below the non-mutagenized control at a dose of 2–3.5 v%.

The graph for UV does not show a similar progression, as the cells specific response to UV irradiation (photolyase) has been effectively disabled by cultivating cells under a SFG-10 filter.

We conclude that the optimal dosages for the nonlethal induction of point mutations for UV is in the range of 10–50 J/m^2^ and either 0.1 or 1 v% for MMS. These dosages induce the maximal rate of non-lethal point mutations, increasing it well above its natural level without creating strong aberrations from the wildtype, such as the loss of the ability to grow on plate. These dosages should therefore be optimal for applications involving directed evolution methods, such as adaption of strains to new culturing conditions.

These determined optimal dosages were then used to effectively increase the thermal tolerance of *Synechocystis* PCC6803. In four rounds of mutagenesis the temperature tolerance under given cultivation conditions was increased from <43°C to 45°C. As the control-culture was not exposed to the mutagens and was not able to show similar increases, it is clear that the increased rate of mutation induced by these mutagens is necessary for such fast achievements.

Two different mutagens were used, which as discussed earlier have differing probabilities to induce the various possible mutations. By using two different mutagens the inherent bias to each mutagen for the induction of certain mutations is reduced, and the chance to induce the optimal mutations should be increased.

A viability analysis of cultures after mutagenesis (fig. 3) clearly shows that from an equal starting pool of healthy cells the non-mutagenized ones died faster and in greater amount than the mutagenized cells when cultivated under selective conditions. This is especially relevant when considering, that at the applied dosage the mutagen itself already induced a high rate of lethality to the culture. For UV the determined survival rate (on plates) at the applied dosages is about 10%, giving a lethality of 90%. Still, the mutagenized cells were faster to recover from the selective conditions than the untreated control, showing that the positive effects of some point mutations outweighed the negative effects such as lethal- and other disadvantageous mutations. The lowest percentage of viable cells reached for the culture was 4%; 19 days after mutagenesis. Of the around 10% of cells which did not develop a lethal mutation because of the treatment, probably only very few have a selective advantage at the increased temperature. These however have had ample time to reproduce until they constitute 4% of the total cells at day 19. Note that the actual number of viable cells in liquid media might be somewhat higher, as lethality was determined by plating.

It is important to note that the improved culture is a mixture of various new strains with an as of yet unknown genetic makeup. This mix however does not have a strongly aberrant phenotype (aside from the thermal tolerance), and many cells are still able to grow on plates. In comparison to methods where single cells are picked directly after mutagenesis before being characterized [24], [25] this presents a slight disadvantage when genetically characterizing the created strains. It does however have the advantage of selecting for cells under the condition where increased growth is desired (*i.e.* liquid media vs plates; day-night cycle etc.). Also a huge pool of candidate mutants (5*10^9^–5*10^10^ in our setup) are cultivated (and characterized) cheaply in a single reactor, where they compete by outgrowing each other, thereby increasing the possibility of finding performant mutants. When isolating single clones from the beginning the number of mutants that are compared is limited by the availability of cultivation vessels under selective conditions.

Currently work is ongoing to isolate monoclonal colonies from the final mutagenized culture. These monoclonal strains will then be screened and characterized for their growth characteristics, before being used for further genetic analysis.

### Conclusions

The determined optimal dosages for the nonlethal induction of point mutations for UV (10–50 J/m^2^) and 0.1/1 v% for MMS appear to be well suited to the generation of tolerances in *Synechocystis* PCC6803.

This was successfully demonstrated by raising *Synechocystis* temperature tolerance by about 2°C in four rounds of mutagenesis. Coupled with an appropriate screening, the described methods should be applicable to induce a variety of desirable characteristics in various strains. These methods are best suited to adaptations which allow an *in vivo* selection, such as adaptations to cultivation conditions (e.g. toxic substances, salt concentration, mixing conditions … ). This is especially attractive for systems where it is not obvious which genes require modifications.

Coupling random mutagenesis with high-throughput screening methods would additionally allow to select for strains with characteristics which do not yield a higher fitness and can not be selected for *in vivo* (eg. fatty acid concentration).

At the very least, random mutagenesis methods can be used as a first step in a combined approach with full genome sequencing (which is now cheaply available through next-generation techniques), to determine suitable target-genes for more focused and comprehensive methods.

## Supporting Information

Additional File S1 **ImageJ plugin for automated colony counting and example pictures.** The plugin can be used for automated counting of cyanobacterial colonies on agar plates using phycocyanin fluorescence. ImageJ is required and can be downloaded from http://rsbweb.nih.gov/ij/download.html. For an installation of the plugin extract the.jar file into the plugin folder and restart ImageJ. The plugin can be found under Plugins > CountCyanoPlate. Example images for the plugin can be found in the folder ExampleImages. The plugin is also hosted at Github at: https://github.com/KatjaSchulze/CyanoColonyCounter
(ZIP)
