# Supplementary figures and images for: The Optimal Mutagen Dosage to Induce Point-Mutations in Synechocystis sp. PCC6803 and Its Application to Promote Temperature Tolerance
Source: PLoS One. 2012 Nov 21;7(11):e49467. doi: 10.1371/journal.pone.0049467 (PMC3504032; doi:10.1371/journal.pone.0049467)

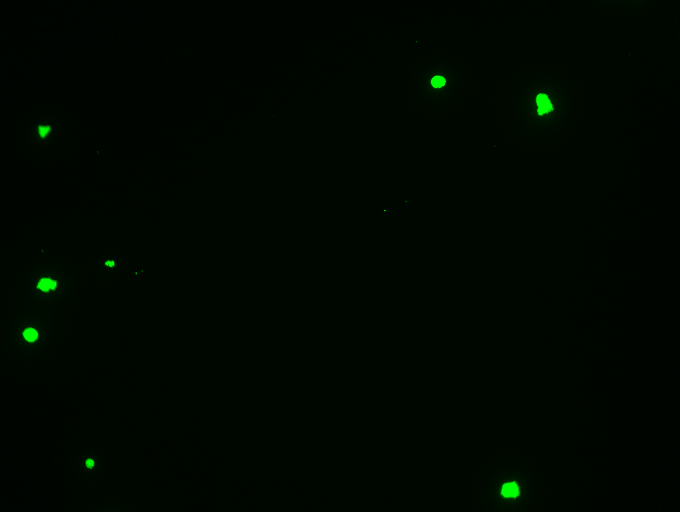

Supplement: Additional File S1 — ImageJ plugin for automated colony counting and example pictures. The plugin can be used for automated counting of cyanobacterial colonies on agar plates using phycocyanin fluorescence. ImageJ is required and can be downloaded from http://rsbweb.nih.gov/ij/download.html. For an installation of the plugin extract the.jar file into the plugin folder and restart ImageJ. The plugin can be found under Plugins > CountCyanoPlate. Example images for the plugin can be found in the folder ExampleImages. The plugin is also hosted at Github at: https://github.com/KatjaSchulze/CyanoColonyCounter (ZIP) [file pone.0049467.s001.zip › ExampleImages/plate5.BMP]

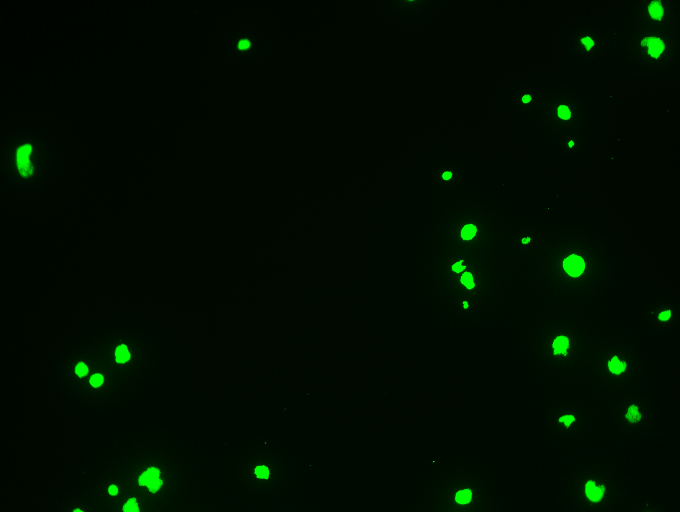

Supplement: Additional File S1 — ImageJ plugin for automated colony counting and example pictures. The plugin can be used for automated counting of cyanobacterial colonies on agar plates using phycocyanin fluorescence. ImageJ is required and can be downloaded from http://rsbweb.nih.gov/ij/download.html. For an installation of the plugin extract the.jar file into the plugin folder and restart ImageJ. The plugin can be found under Plugins > CountCyanoPlate. Example images for the plugin can be found in the folder ExampleImages. The plugin is also hosted at Github at: https://github.com/KatjaSchulze/CyanoColonyCounter (ZIP) [file pone.0049467.s001.zip › ExampleImages/plate4.BMP]

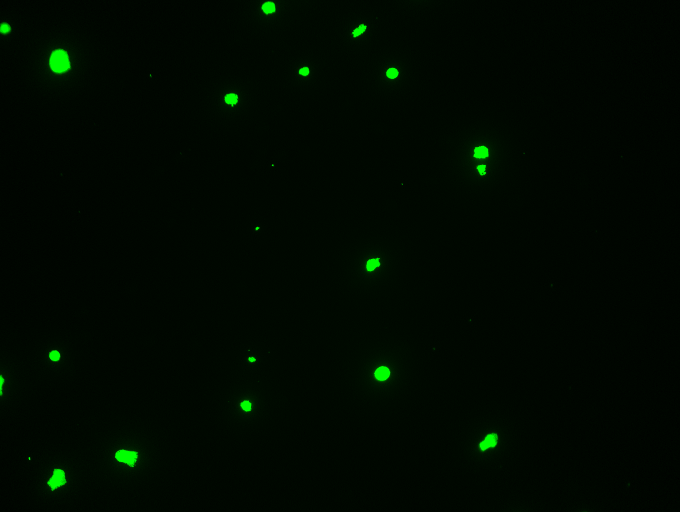

Supplement: Additional File S1 — ImageJ plugin for automated colony counting and example pictures. The plugin can be used for automated counting of cyanobacterial colonies on agar plates using phycocyanin fluorescence. ImageJ is required and can be downloaded from http://rsbweb.nih.gov/ij/download.html. For an installation of the plugin extract the.jar file into the plugin folder and restart ImageJ. The plugin can be found under Plugins > CountCyanoPlate. Example images for the plugin can be found in the folder ExampleImages. The plugin is also hosted at Github at: https://github.com/KatjaSchulze/CyanoColonyCounter (ZIP) [file pone.0049467.s001.zip › ExampleImages/plate3.BMP]

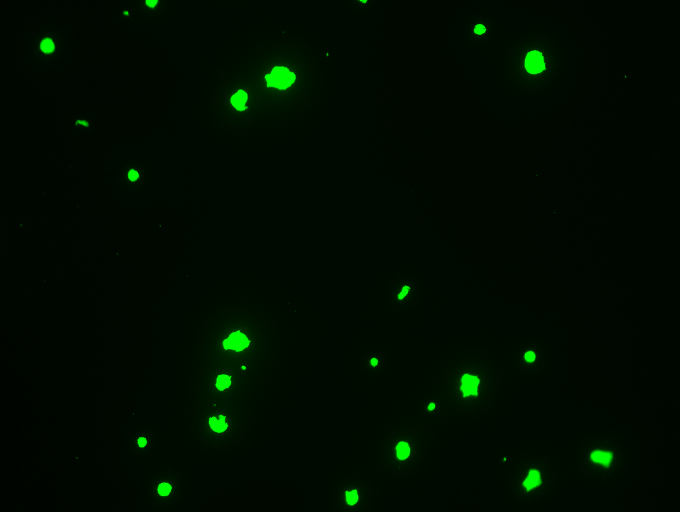

Supplement: Additional File S1 — ImageJ plugin for automated colony counting and example pictures. The plugin can be used for automated counting of cyanobacterial colonies on agar plates using phycocyanin fluorescence. ImageJ is required and can be downloaded from http://rsbweb.nih.gov/ij/download.html. For an installation of the plugin extract the.jar file into the plugin folder and restart ImageJ. The plugin can be found under Plugins > CountCyanoPlate. Example images for the plugin can be found in the folder ExampleImages. The plugin is also hosted at Github at: https://github.com/KatjaSchulze/CyanoColonyCounter (ZIP) [file pone.0049467.s001.zip › ExampleImages/plate2.BMP]

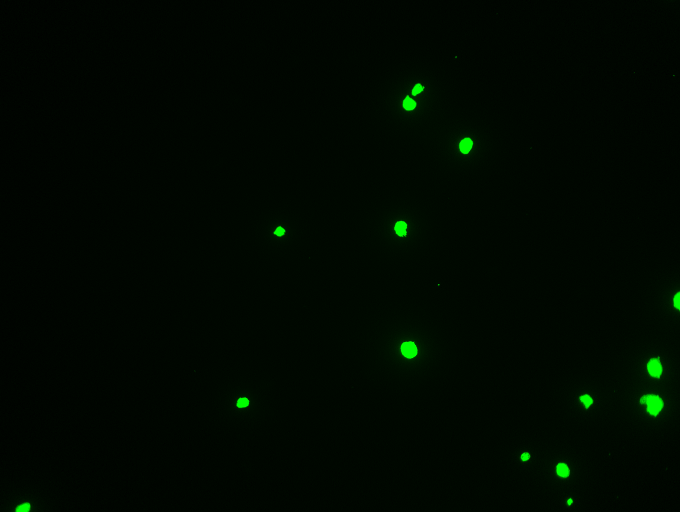

Supplement: Additional File S1 — ImageJ plugin for automated colony counting and example pictures. The plugin can be used for automated counting of cyanobacterial colonies on agar plates using phycocyanin fluorescence. ImageJ is required and can be downloaded from http://rsbweb.nih.gov/ij/download.html. For an installation of the plugin extract the.jar file into the plugin folder and restart ImageJ. The plugin can be found under Plugins > CountCyanoPlate. Example images for the plugin can be found in the folder ExampleImages. The plugin is also hosted at Github at: https://github.com/KatjaSchulze/CyanoColonyCounter (ZIP) [file pone.0049467.s001.zip › ExampleImages/plate1.BMP]
